# Worldwide co-occurrence analysis of 17 species of the genus *Brachypodium* using data mining

**DOI:** 10.7717/peerj.6193

**Published:** 2019-01-14

**Authors:** Simon Orozco-Arias, Ana María Núñez-Rincón, Reinel Tabares-Soto, Diana López-Álvarez

**Affiliations:** 1 Department of Electronics and Automatization, Universidad Autónoma de Manizales, Manizales, Colombia; 2 Centro de Bioinformática y Biología Computacional de Colombia BIOS, Manizales, Colombia; 3 Facultad de Ciencias Agropecuarias, Universidad Nacional de Colombia, Palmira, Colombia

**Keywords:** Data mining, Co-occurrence analysis, Association rules, Bioinformatics, *Brachypodium*

## Abstract

The co-occurrence of plant species is a fundamental aspect of plant ecology that contributes to understanding ecological processes, including the establishment of ecological communities and its applications in biological conservation. A priori algorithms can be used to measure the co-occurrence of species in a spatial distribution given by coordinates. We used 17 species of the genus *Brachypodium*, downloaded from the Global Biodiversity Information Facility data repository or obtained from bibliographical sources, to test an algorithm with the spatial points process technique used by Silva et al. (2016), generating association rules for co-occurrence analysis. *Brachypodium* spp. has emerged as an effective model for monocot species, growing in different environments, latitudes, and elevations; thereby, representing a wide range of biotic and abiotic conditions that may be associated with adaptive natural genetic variation. We created seven datasets of two, three, four, six, seven, 15, and 17 species in order to test the algorithm with four different distances (1, 5, 10, and 20 km). Several measurements (support, confidence, lift, Chi-square, and *p*-value) were used to evaluate the quality of the results generated by the algorithm. No negative association rules were created in the datasets, while 95 positive co-occurrences rules were found for datasets with six, seven, 15, and 17 species. Using 20 km in the dataset with 17 species, we found 16 positive co-occurrences involving five species, suggesting that these species are coexisting. These findings are corroborated by the results obtained in the dataset with 15 species, where two species with broad range distributions present in the previous dataset are eliminated, obtaining seven positive co-occurrences. We found that *B. sylvaticum* has co-occurrence relations with several species, such as *B. pinnatum*, *B. rupestre*, *B. retusum*, and *B. phoenicoides*, due to its wide distribution in Europe, Asia, and north of Africa. We demonstrate the utility of the algorithm implemented for the analysis of co-occurrence of 17 species of the genus *Brachypodium*, agreeing with distributions existing in nature. Data mining has been applied in the field of biological sciences, where a great amount of complex and noisy data of unseen proportion has been generated in recent years. Particularly, ecological data analysis represents an opportunity to explore and comprehend biological systems with data mining and bioinformatics tools.

## Introduction

The analysis of species co-occurrence patterns has a long history in ecology since the 1970s, and has played a central role in debates about the importance of competition in structuring ecological communities, environmental conditions, and the existence of assembly rules (Veech, 2013). In the early days of ecology the interactions among species have been of considerable interest for ecologists (Saiz & Alados, 2011). Regardless of the usual analysis used in co-occurrence studies which are based on analyzing pairs of species instead of entire matrices. The future of studies in this field should attempt to make a priori predictions of the species pairs that should be classified positively, negatively or randomly associated, according to theory or to the hypothesis testing (Veech, 2013). There are many methods to measure the co-occurrence of species in a spatial distribution using the information of coordinates, including neutral models of diversity (Trejo-Barocio & Arita, 2013), codispersion analysis to characterize spatial patterns in co-occurrence (Buckley, Case & Ellison, 2016), and Spatial Points Processes (SPP) used by Silva et al., (2016), on which we based this study. SPP are defined as a set of observations (*X*^1^, *X*^2^,…, *X*^n^) within a study area, where each point has at least a pair of coordinates (Lloyd, 2006). SPP analyzes the spatial structure rather than its variation in space, and can also infer spatial associations in an univariate (one point process equals one species), or bivariate spatial points process (two different points processes, i.e., two species). In plant ecology, Ripley’s K-function (Ripley, 1977) is commonly used to detect the spatial distribution of individuals within communities and the underlying processes controlling the observed patterns (Silva et al., 2016).

For this study, we used the genus *Brachypodium* as input to establish the co-occurrence of several species of this genus using the algorithm implemented in Silva et al. (2016). Further, we applied data mining on georeferenced data for the species. In the last decade, *Brachypodium* spp. has emerged as an effective model for monocot species (Catalan et al., 2015; Fitzgerald et al., 2015). *Brachypodium* is a relatively small genus that contains around 18 species distributed worldwide (Catalan et al., 2012; Catalan & Olmstead, 2000; Schippmann, 1991). According to the most recent taxonomic update, three of them are annual species and 15 are perennial taxa (Woods & Amasino, 2015). Annual and perennial species have a large distribution (Table 1 of this study), along the circum-Mediterranean and Eurasian region, America (from Mexico to Peru–Bolivia), Asia (Taiwan, Malasia), and Africa (Madagascar, Tropical Africa and South Africa) (Catalan et al., 2015; López-Alvarez et al., 2015; Schippmann, 1991).

**Table 1:** Basic parameter values used in the implementation of the algorithm.

Variable	Value
Minimum support	0.15
Minimum confidence	0.3
Negative minimum lift	1
Positive minimum lift	1

We used an a priori algorithm as part of unsupervised techniques used by data mining that discovers relations on their own, not relying on prior knowledge and used clustering to detect similarities (Kropp, 2004). The aim of the a priori algorithm is to resolve the problem of finding association rules within the purview of database mining, detecting all item sets that have transaction support above the minimum support. The support for an item set is the number of transactions that contains the item set (Kropp, 2004), where each transaction is composed of all specimens within the specified distance of a given specimen (Silva et al., 2016). A transaction in data mining is a set of items that share some condition, such as a sale transaction, which contains products bought by a costumer. The algorithm first counts occurrences for determining largest item sets, then generates candidates using the apriori-gen function described by Agrawal & Srikant (1994). Finally, the database is scanned and the support of each candidate is calculated to add candidates that satisfy parameters given to create association rules. The goal of association rules is to discover recurring items from a set of transactions, deriving rules from associations between the items involved in each transaction without implying causality. A logical statement between two items is the exemplification of a rule, for example, given the species *B. hybridum* analyzed as an antecedent and the species *B. distachyon* as the consequent (*B. hybridum* → *B. distachyon*), a pattern where *B. hybridum* and *B. distachyon* appear together can be understood.

In the present research, we analyze the spatial associations among 17 *Brachypodium* species in a broad distribution, with the aim of (1) testing co-occurrence using the algorithm proposed by Silva et al. (2016), taking into account that 12 of the 17 species used are distributed between the Eurasian and circum-Mediterranean region (western Mediterranean, eastern Mediterranean, western Eurasia (from Atlantic to Urals), eastern Eurasia (from Urals to Pacific), and Eastern Asia, Canary Isles), so it is likely that some of them are coexisting in their native distribution areas and these results could be contrasted with species from other continents with specific distributions (three species from Africa, one from America, and one from Asia) (2) analyzing the negative, positive, and neutral rules created by the a priori algorithm, and (3) demonstrating that this kind of study can be implemented with any data available in the Global Biodiversity Information Facility (GBIF) that contains latitude and longitude data.

## Materials and Methods

### Implementation of the algorithm in Python

The methodology presented in Silva et al. (2016) was implemented in Python 2.7 followed by the Algorithm 1 (Silva et al., 2016), (https://github.com/simonorozcoarias/co-ocurrence_analysis). Two parameters were needed to create a transactions file, the geo-located data with the following structure: species_name, latitude, longitude and distance in meters. The first step for the algorithm was to create a folder named “DE_results” with the output files (transactions.csv, all_rules.txt, positive_rules.txt, negative_rules.txt), using all records of the data file. The distance between all records was calculated, one by one, based on its latitude and longitude using the formula of Haversine (Chopde & Nichat, 2013). If the distance was lower than a parameter (1, 5, 10, or 20 km), a transaction was created and stored in the transaction file. Each transaction was composed by the name of the species involved in it, separated by a comma.

The second step was to define the basic values of parameters such as minimum support, minimum confidence, negative minimum lift, and positive minimum lift (Table 2), which lift is the measure of importance of a rule. Next, rules were generated through the a priori algorithm presented in Agrawal & Srikant (1994), using the transaction file previously created. These rules are computed from the data and, unlike the if-then rules of logic, association rules are probabilistic in nature. This algorithm created a set of rules, where each rule contained antecedent (if) and consequent (then) elements and a confidence value, that is, if the algorithm creates the following association rule species X ⇒ species Y with confidence 0.5 indicates that species X and Y are usually found together and the confidence value 0.5 show us that if you find species X there is a 50% chance to find species Y. In addition, the frequency of each element of the rule was calculated using the Chi-squared function from the scipy.stats python package (Woods & Amasino, 2015). This function calculated the Chi-squared and *p*-value for the rule. We calculated support and lift values from definitions presented in Silva et al. (2016). We considered a rule as positive if it had a *p*-value equal to ∼0, and a rule as negative if it had a *p*-value equal to ∼1. Positive and negative rules were stored in the all-rules file.

**Table 2:** Datasets specification using *Brachypodium* species.

Dataset	Species	No. species	Total records
Dataset 1	*B. sylvaticum, B. rupestre*	2	105,986
Dataset 2	*B. stacei, B. distachyon, B. hybridum*	3	325
Dataset 3	*B. mexicanum, B. bolusii, B. retusum, B. rupestre*	4	26,018
Dataset 4	*B. genuense, B. phoenicoides, B. retusum, B. rupestre, B. pinnatum, B. sylvaticum*	6	177,685
Dataset 5	*B. genuense, B. phoenicoides, B. retusum, B. rupestre, B. glaucovirens, B. pinnatum, B. sylvaticum*	7	177,691
Dataset 6	*B. arbuscula, B. boissieri, B. flexum, B. genuense, B. hybridum, B. kawakamii, B. madagascariense, B. mexicanum, B. bolusii, B. phoenicoides, B. retusum, B. rupestre, B. distachyon, B. glaucovirens, B. stacei*	15	35,697
Dataset 7	*B. arbuscula, B. boissieri, B. flexum, B. genuense, B. hybridum, B. kawakamii, B. madagascariense, B. mexicanum, B. bolusii, B. phoenicoides, B. retusum, B. rupestre, B. distachyon, B. glaucovirens, B. pinnatum, B. stacei, B. sylvaticum*	17	179,026

In this study was used the following set of measures to evaluate the quality of the rules generated by the algorithms of association rules: support, confidence, lift (Han & Kamber, 2006), Chi-square (Hahsler, Grün & Hornik, 2005), and *p*-value (Liu, Zhang & Wong, 2011). The first two measures of support and confidence were used to define the species’ pairs and groups; the third (lift) assesses the association type (positive or negative), and the Chi-square and *p*-value measures consider the degree of independence of the species. For example, considering two species, *B. retusum* and *B. phoenicoides*, the support is the probability P of transactions with both species and is defined as support (*B. retusum* → *B. phoenicoides*) = P (*B. retusum* ∪ *B. phoenicoides*). The confidence is defined as the frequency with which items are found in the transaction *B. retusum* containing *B. phoenicoides*, and is defined as the conditional probability confidence (*B. retusum* → *B. phoenicoides*) = P (*B. retusum* | *B. phoenicoides*). The lift is the measure of the importance of a rule and can be defined by P (*B. retusum* ∪ *B. phoenicoides*)/(P (*B. retusum*) ∗ P (*B. phoenicoides*)) (Silva et al., 2016). The Chi-squared of a rule may be computed directly from the values of confidence, support, and lift (interest) of the rule in question (Alvarez, 2003). In the case of association rules the *p*-value of a rule, R = (*B. retusum* → *B. phoenicoides*), is defined as the probability of observing R or a rule more extreme than R, given that the two sides of R are independent. If a rule R has low *p*-value, then R has a low chance of occurring if its two sides are independent (Liu, Zhang & Wong, 2011).

### Creation of the dataset for the evaluation of co-occurrence

The previously implemented algorithm was executed with seven different datasets, which contained a diverse amount of records and species (Table 3). We chose to analyze different combinations of species to ensure that the co-occurrences established have consistency and were not affected by the number of species involved or by the number of records. Whereby, we used two, three, and four species (datasets 1, 2, and 3) with the aim of identifying the minimum number of species possible for implementing the algorithm and to discover positive rules, considering that the dataset contained a number of diverse records and distributions.

**Table 3:** Description of the native distribution of the 17 *Brachypodium* species used in datasets.

Name	Native distribution	Records
*Brachypodium genuense*	Italy	11
*Brachypodium phoenicoides*	West Mediterranean	8,908
*Brachypodium pinnatum*	Eurasia, SW Asia	40,552
*Brachypodium retusum*	Mediterranean	22,228
*Brachypodium rupestre*	West Eurasia	3,209
*Brachypodium sylvaticum*	PanEurasia (Eurasia, Macaronesia)	102,777
*Brachypodium distachyon*	Circum-Mediterranean (Mediterranean, SW Asia)	119
*Brachypodium stacei*	Circum-Mediterranean (Mediterranean, Macaronesia, SW Asia)	40
*Brachypodium hybridum*	Circum-Mediterranean (Mediterranean, Macaronesia, SW Asia)	166
*Brachypodium arbuscula*	Macaronesia: Canary isles (Spain)	17
*Brachypodium boissieri*	Spain: Betic mountain ranges (southern Spain)	198
*Brachypodium flexum*	Tropical Africa and South Africa	105
*Brachypodium kawakamii*	Taiwan	107
*Brachypodium madagascariense*	Madagascar	2
*Brachypodium mexicanum*	America (from Mexico to N Bolivia)	533
*Brachypodium bolusii*	South Africa	49
*Brachypodium glaucovirens*	East Mediterranean and SW Asia	6
Total		179,026

In total we used 17 species of the genus *Brachypodium*, downloaded from the GBIF data repository (http://www.gbif.org) or obtained from bibliography sources (López-Alvarez et al., 2015; Schippmann, 1991). All datasets can be downloaded at https://github.com/simonorozcoarias/co-ocurrence_analysis/tree/master/raw_data. All of the data used in this study was validated and filtered according to each species and its native distribution (Table 1). In addition, each dataset was tested with four different distances (1, 5, 10, and 20 km), due to the cosmopolitan distribution of the genus *Brachypodium*, since some species have very specific distributions and it is necessary to establish their co-occurrence at different distances.

## Results

We observed the importance of the amount of transactions generated in the datasets, in addition to the number of species in each transaction (Supplemental Material 1); this is due to the creation of the transactions according to the distances between the registers. No negative rules were created in all datasets (Table 4).

**Table 4:** Results of all itemsets with four different distances.

Itemsets	Distance (km)	Total transactions	Unique transactions	All rules	Positive rules
Dataset 1 (two species)	1	4,666	2	2	0
	5	6,699	2	2	0
	10	10,878	2	2	0
	20	19,454	2	2	0
Dataset 2 (three species)	1	27	7	4	0
	5	39	7	4	0
	10	52	7	5	0
	20	109	9	5	0
Dataset 3 (four species)	1	943	2	2	0
	5	1,265	2	2	0
	10	1,826	2	2	0
	20	2,607	2	2	0
Dataset 4 (six species)	1	111,891	72	4	4
	5	128,843	72	4	4
	10	148,157	72	6	6
	20	160,731	71	16	16
Dataset 5 (seven species)	1	111,892	75	4	4
	5	128,848	77	4	4
	10	106,851	75	4	4
	20	160,735	86	16	16
Dataset 6 (15 species)	1	23,149	41	2	0
	5	26,039	77	2	0
	10	30,082	110	2	0
	20	32,490	121	7	7
Dataset 7 (17 species)	1	112,321	118	4	4
	5	129,566	207	4	4
	10	148,972	273	6	6
	20	161,475	281	16	16

Dataset with few species (2, 3, and 4) did not generate any positive rules. For dataset 1, we found the relation between the two species, taking into account a support value of 1 for all the distances, resulting in a co-existence of 100% in the generated transactions. For example, at a distance of 20 km, the species coexist on 19,454 occasions. On the other hand, by looking at the confidence results, we could determine that *B. sylvaticum* was found when *B. rupestre* was present in 100% of the cases and vice versa. However, evaluating the scores for lift and Chi-square (Supplemental Material 1), we found that there is no independence between both species. In dataset 2, despite not finding positive rules for the co-occurrences of the three species evaluated, we found four rules generated for one and five km distances, while for 10 and 20 km we found five completely different rules (Supplemental Material 1). At one and five km distances, the support value for all the transactions was 0.5, meaning that the species coexist 50% of the time. In the case of the 10 km distance, all the transactions were 0.6, with the exception of the transaction *B. distachyon, B. stacei* vs. *B. hybridum* that represented a coexistence of 20%. Finally, for the 20 km distance the support value was 0.4, except for the relationship between *B. distachyon* vs. *B. stacei* with a score of 0.2. By analyzing the confidence value of *B. hybridum*, we found that *B. distachyon* was present in 70%, 61%, 57%, and 52% (1, 5, 10, and 20 km, respectively) of the cases, while *B. distachyon* was found when *B. hybridum* was present in 100% of the cases, except for the 20 km distance (96%). *B. hybridum* was found when *B. stacei* was present in 41%, 49%, 58%, and 63% (following the same distances). In the opposite case, *B. stacei* was found when *B. hybridum* was present in 100% of the transactions, excepting for the 20 km distance (97%). In the case of the 10 km distance, we found *B. stacei* and *B. distachyon* when *B. hybridum* was present 100% of the time. However, for the 20 km distance, *B. distachyon* was found when *B. stacei* happened to be present only in 31% of the cases. For the dataset 3 (four species), we observed 100% in support and confidence values for all four distances when the species *B. rupestre* and *B. retusum* were used in all the transactions.

For datasets 4, 5, and 7, we found 30 rules with identical species, with equal or very close values of support and confidence; for this reason we analyzed these results together. The only exception was dataset 5 that had 28 rules. Dataset 4 included six species of the genus *Brachypodium*, dataset 5 contained seven species, and dataset 7 had data from the 17 species used in the study. In the three datasets we found that at the one and five km distances the same four rules were generated between *B. retusum* vs. *B. phoenicoides* and *B. sylvaticum* vs. *B. pinnatum*, all having the same support value of 0.5, meaning a coexistence of 50%. In terms of their confidence values, we discovered that *B. retusum* was found when *B. phoenicoides* was present 93% of the time, and 90% in the opposite case. For *B. sylvaticum*, we found that *B. pinnatum* was present 84% of the time, while the other way around there was a 99% of probability. At the 10 km distance for datasets 4 and 7, the following rules were created: *B. phoenicoides* vs. *B. sylvaticum* having a support value of 0.16 and the confidence value revealed that *B. phoenicoides* was found when *B. sylvaticum* was present 68% of the times. Another rule was *B. retusum* vs. *B. sylvaticum* with the same value of support, but *B. retusum* was found when *B. sylvaticum* was present 67% of the times. In the case of the 20 km distance, the same 16 rules were obtained for all three datasets, of which six rules were those stated above, eight were three species transactions, and two were: *B. rupestre* vs. *B. sylvaticum* and *B. rupestre* vs. *B. pinnatum*. The *B. rupestre* vs. *B. sylvaticum* rule has a support value of 0.25 and according to the confidence report *B. rupestre* was found when *B. sylvaticum* was present 99% of the times. On the other hand, *B. rupestre* vs. *B. pinnatum* has the same support value, but *B. rupestre* was found when *B. pinnatum* was present 97% of the cases.

Additionally, we found *B. retusum* vs. *B. sylvaticum* and *B. phoenicoides* with a support value of 0.31 and a confidence score of 76%, as the probability of finding *B. retusum* when *B. sylvaticum* and *B. phoenicoides* were present. In the case of *B. phoenicoides* vs. *B. retusum* and *B. sylvaticum* the support value was the same, but the probability of finding *B. phoenicoides* when *B. retusum* and *B. sylvaticum* were present was of 73%. For the rule *B. retusum* and *B. phoenicoides* vs. *B. sylvaticum* the support value was the same and the confidence value was 78%. The rule *B. retusum* and *B. sylvaticum* vs. *B. phoenicoides* had a support value of 0.31 and the probability of finding *B. retusum* and *B. sylvaticum* when *B. phoenicoides* was present was of 96%, the highest value of all reported rules with these three species. The *B. sylvaticum* and *B. phoenicoides* vs. *B. retusum* rule had a confidence value of 92% and a support value of 0.31. The following rules had a support value of 0.18; *B. rupestre* vs. *B. sylvaticum* and *B. pinnatum* had coexistences of 97%; *B. sylvaticum* and *B. rupestre* vs. *B. pinnatum* showed coexistences of 98%; and *B. pinnatum* and *B. rupestre* vs. *B. sylvaticum* had a 99% probability that the two species were found when *B. sylvaticum* was present.

Regarding the dataset 6 that included 15 species and exclude two of broad range distribution (*B. sylvaticum* and *B. pinnatum*), the species, *B. retusum* and *B. phoenicoides* were the only related in 1, 5, and 10 km distances coexisting in a 100% of the rules generated with the above stated distances. Also, *B. retusum* was found when *B. phoenicoides* was present in 98% of the cases. The same behavior was found in the coexistence relationship of *B. phoenicoides* vs. *B. retusum*. We found a different behavior using the 20 km distance, like generated rules associating *B. retusum*, *B. phoenicoides*, and *B. hybridum*, where *B. hybridum* and *B. retusum* coexisted in 57% of the transactions, *B. retusum* and *B. phoenicoides* in 71%, and *B. hybridum* vs. *B. phoenicoides* in 57%. Additionally, rules were created where the three species coexisted in 43% of the times. On the other hand, *B. hybridum* was found when *B. retusum* was present in 99% of the cases, *B. retusum* was found when *B. phoenicoides* was present in 98%, and *B. phoenicoides* was found when *B. retusum* was present in 98%. With respect to the three-species rules created, we observed a highly interesting behavior showing that *B. hybridum* was found when *B. retusum* and *B. phoenicoides* were present in 96% of the cases, *B. hybridum* and *B. retusum* were found when *B. phoenicoides* was present in 97%, and *B. hybridum* and *B. phoenicoides* were found when *B. retusum* was present ∼100% of the transactions, proving a strong coexistence between the three species.

We found 16 positive co-occurrences (Supplemental Material 2) using a distance of 20 km in datasets 4, 5, and 7, involving the same five species (*B. retusum, B. phoenicoides, B. rupestre, B. sylvaticum*, and *B. pinnatum*). These results suggest that only these species are coexisting, even if it is increasing the number of other species to correlate in the study. Meanwhile for the dataset 6, we found seven different rules involving only three species. For this reason, we used datasets 6 (15 species) and 7 (17 species) for have different points of comparison the following analysis.

### Exhaustive analysis of positive rules found in datasets with 15 and 17 species

**Figure 1:**
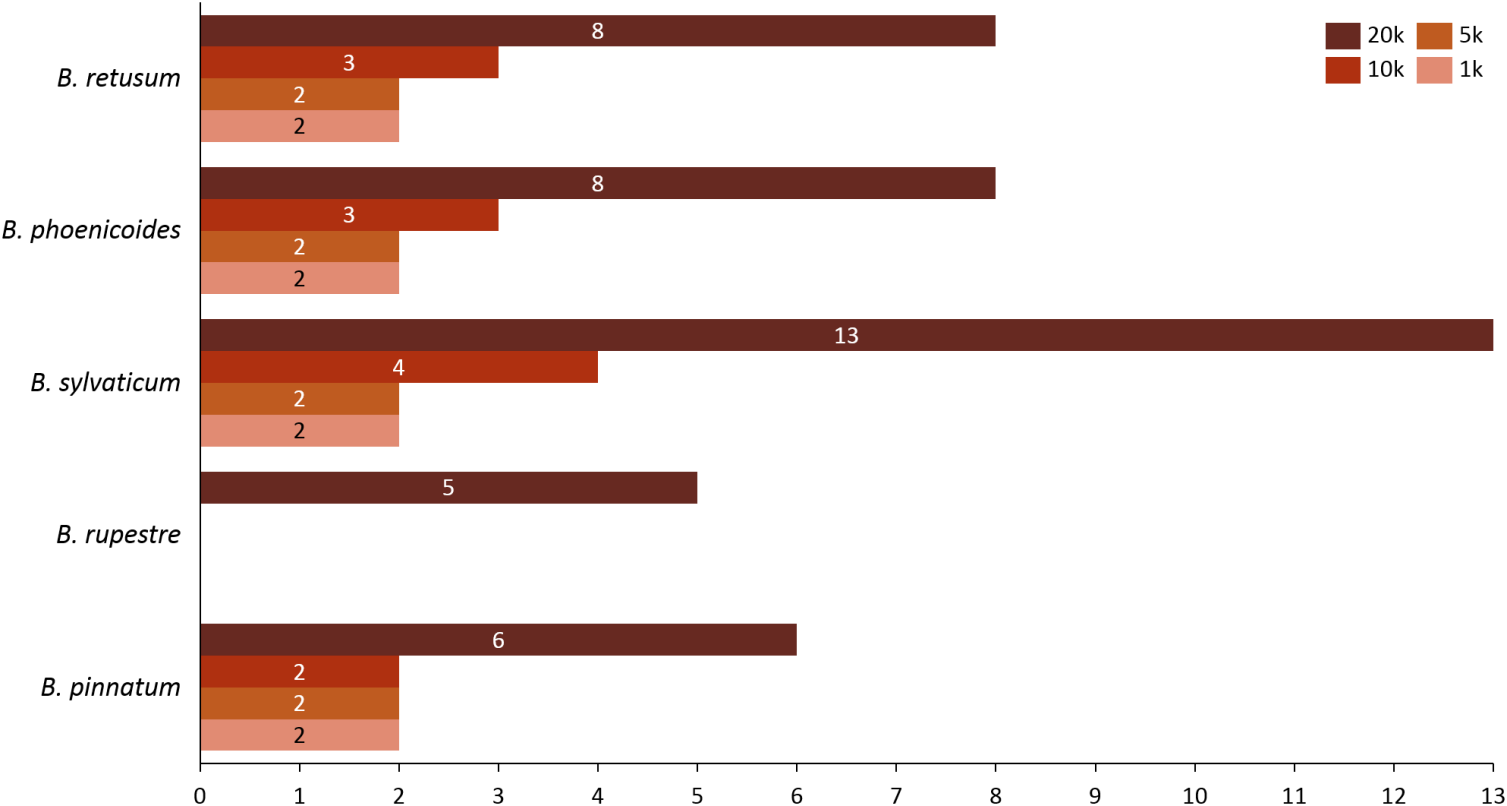
Number of appearances of each species in the rules generated in dataset 7 using all distances.

**Figure 2:**
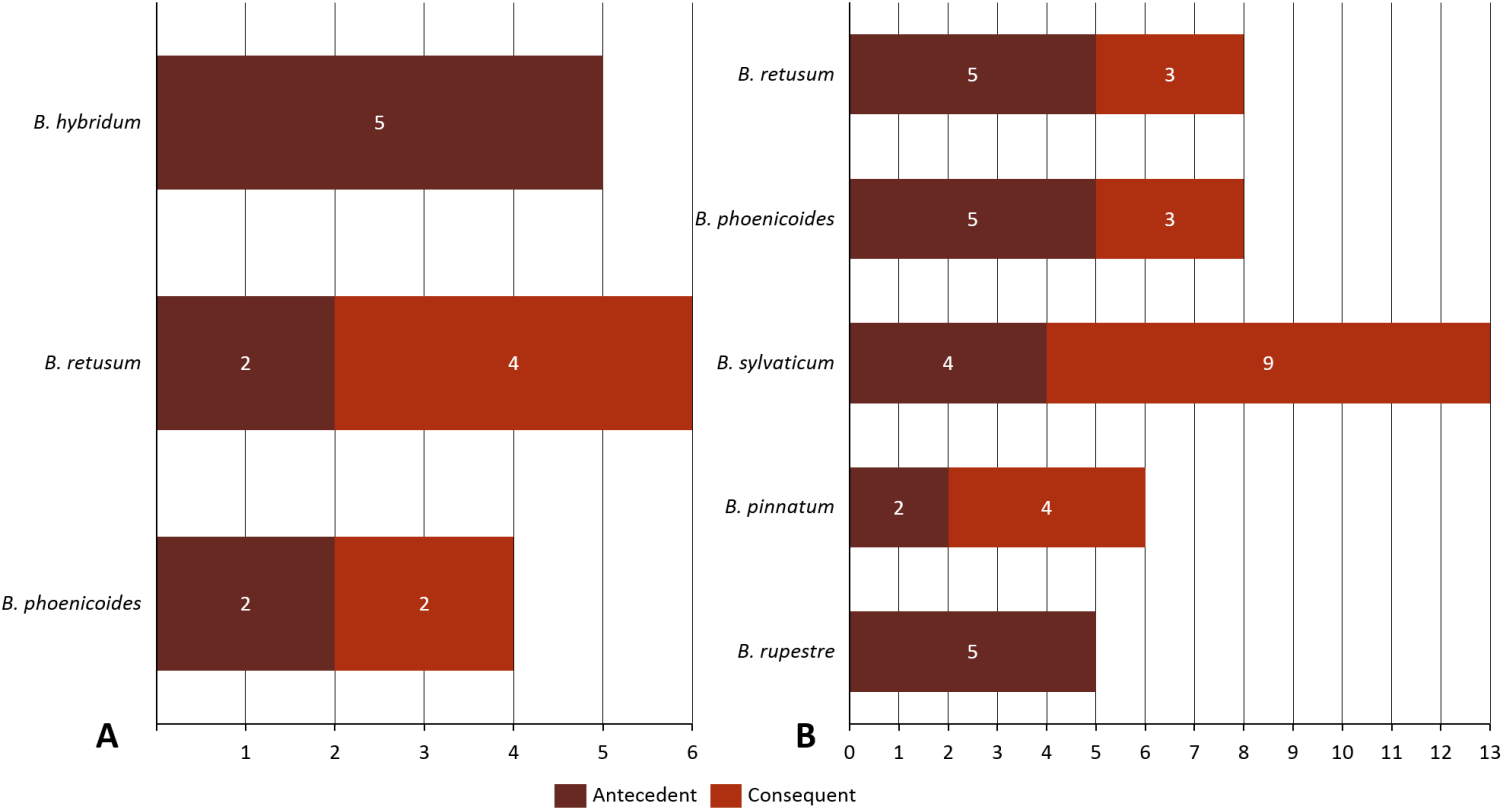
Behavior of species in the rules generated in (A) dataset 6 and (B) dataset 7.

Given the importance of analyzing the coexistence of a large number of species, datasets 6 and 7 acquire relevance in the study, since they bring us a little closer to a real situation in nature. For dataset 6, taking into account a distance of 20 km, we found only three species present in the rules; *B. hybridum* in five rules, *B. phoenicoides* and *B. retusum* in six rules. On the contrary, no positive rules were generated with the other distances. Nonetheless, by analyzing dataset 7 we found that using distances of 1, 5, and 10 km, the rules generated were composed of only four species, but with 20 km the rules were composed of five species, where the new species corresponds to *B. rupestre* (Fig. 1). Also, we analyzed the role for each species in their positive rules (as antecedent or consequent) using the 20 km distance for datasets 6 and 7 (Fig. 2).

We observed that as the tested distance increased so did the complexity of rules. Using distances such as 1, 5, and 10 km rules with only two species were generated, but with 20 km there appeared rules that were composed of three species. After creating the rules, the algorithm filtered them using the lift value in order to classify them as positive, negative, independent, or negligible. We found a relation of co-occurrences between some species contained within positive rules generated, and compared those relations with non-filtered generated rules.

Finally, we found only one difference between species that were contained in datasets 6 and 7, *B. hybridum* appeared just in the first. Using the number of appearances of each species in the created transactions and its relation with the number of total transactions, we found the importance that the amount of registries have in the creation of positive rules and its impact on other transactions.

Species distribution models quantify the relationship between species and their environments without considering the possible biotic interactions (Pollock et al., 2014). Therefore, not all the features that influence species co-occurrence will be captured by environmental variables. Accordingly, other alternatives to quantify co-occurrence can provide insights into the underlying causes of similarities and dissimilarities in distributions among species.

In the natural habitats and distributions of the genus *Brachypodium*, it is evident that some species are co-occurring. For instance, in the case of annual species such as *B. distachyon*, *B. stacei*, and *B. hybridum* (López-Alvarez et al., 2015) or in the perennial Eurasian species where *B. pinnatum*, *B. rupestre*, and *B. sylvaticum* grow in mesic to humid open grasslands and forests (Catalán et al., 2016; Scholthof et al., 2018). This kind of algorithms is very useful, because it enables us to mathematically check the co-occurrence of biological species. This study allowed us to determine the relationships between the different species of the genus *Brachypodium* using various data sets. We observed that it is necessary to obtain a broad number of generated transactions composed of various species in order to create rules for relations, since with few transactions the *p*-value has no statistical significance (Table 4). Therefore, the algorithm ignores this relation in the positive rule creation process. Table 4 clearly shows this case with datasets 1, 2, and 3.

We found that the coexistence of a small group of species can be evaluated and correlated with deeper studies of ecological niche models, in dataset 2, the coexistence of *B. distachyon, B. stacei*, and *B. hybridum* was of 20% for a distance of 10 km. These results agree with the data reported in the study of López-Alvarez et al. (2015), where the calculated area of *B. hybridum* was 1,464,910 km^2^ and the overlap area of *B. distachyon* and *B. stacei* was of 294,041 km^2^, representing a coexistence of 20%. Using a distance of 20 km, we discovered that *B. distachyon* was found where *B. stacei* was present in 31% of the cases. It is possible to verify this result taking into account data from López-Alvarez et al. (2015), where the overlapping areas of *B. distachyon* and *B. stacei* in addition to the overlapping area of the three species (*B. distachyon*, *B. stacei*, and *B. hybridum*) is 29.6%, as the area where the two species are coexisting. Thus, our results are consistent with those reported by previous studies.

However, by adding more species into the analysis, we wanted to test the potential of the algorithm to estimate the possible correlations that occur between species in their natural habitat. Therefore for the datasets 4, 5, 6, and 7, the rule *B. retusum* vs. *B. phoenicoides* was generated with a support of 0.5 in the first three and with one in the last, demonstrating that these two species are coexisting in a large extension of their distribution, and that its correlation is so high that it is not affected by the amount of additional species being tested in the datasets, helping us to determine that when there is a real correlation between species, these will be reflected in the support of their association. Different researchers have reported that both species can be found together in North Africa and in Western Europe, according to their geographic distribution (Catalan et al., 2015; Schippmann, 1991); even Khan & Stace, (1999), reported the association of *B. phoenicoides* with *B. retusum* rather than with *B. pinnatum*; which was not found in this study either.

The rule *B. sylvaticum* vs. *B. pinnatum* was found in datasets 4, 5, and 7 with a coexisting rate of 50% and confidence values of 0.84 and 0.99 in the opposite direction; recent studies of Díaz-pérez et al., (2018) revealed alleles associated with genomes of these species are present in the allotetraploid *B. phoenicoides*, therefore find coexisting these two species can corroborate and help explain these results. In the same way, genomes of *B. pinnatum* and *B. sylvaticum* together with *B. arbuscula* were potentially involved in the origins of seven allopolyploid core perennial species: *B. phoenicoides, B. kawakamii, B. madagascariense, B. retusum, B. flexum*, and *B. bolusii* (Díaz-pérez et al., 2018). Others correlations found in 20 km for datasets 4, 5 and 7, among these species can be explained perfectly, either because they are their parents, or because it is a child coexisting with their parent, for example, *B. phoenicoides* with *B. pinnatum* or *B. retusum* with *B. sylvaticum*; we also found association of *B. rupestre* with *B. sylvaticum* and *B. pinnatum* that for Díaz-pérez et al., (2018) this specie have alleles associated to the genome of *B. glaucovirens*, but also to *B. sylvaticum, B. pinnatum*, and *B. arbuscula*.

**Figure 3:**
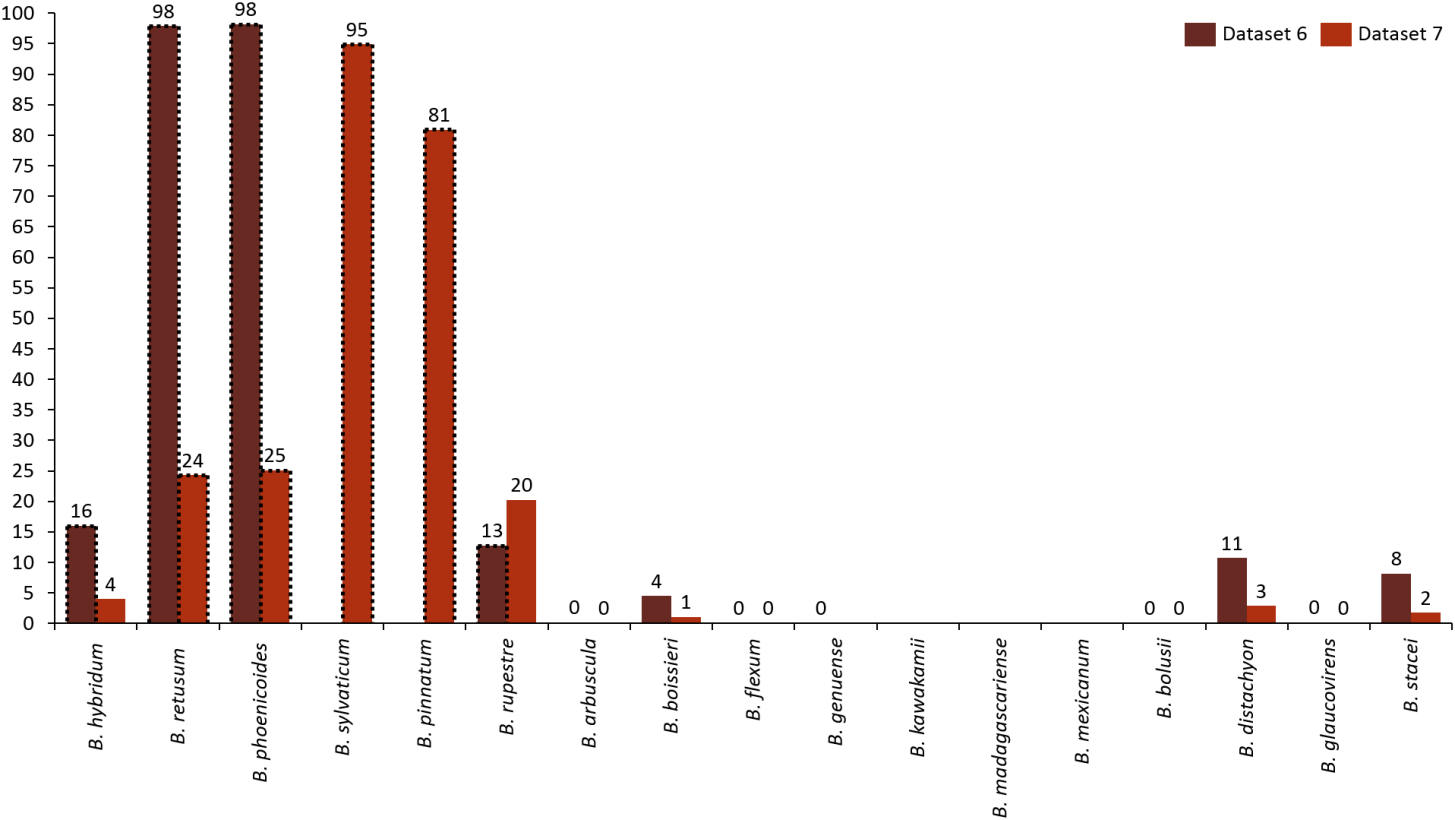
Frequency of transactions created for each species. Species with positive generated rules are presented with dotted lines.

**Figure 4:**
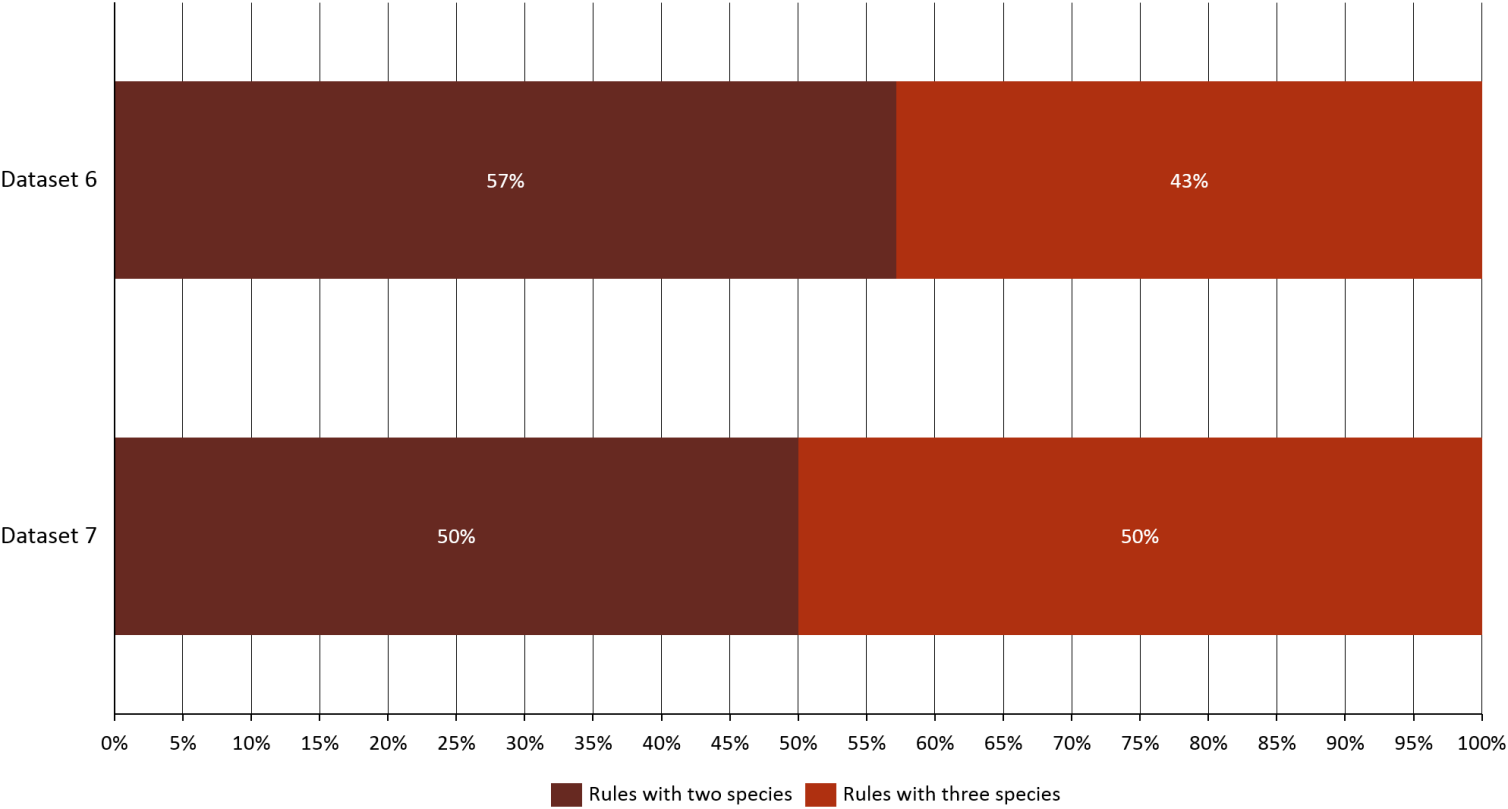
Rules composition and complexity for datasets 6 and 7.

**Figure 5:**
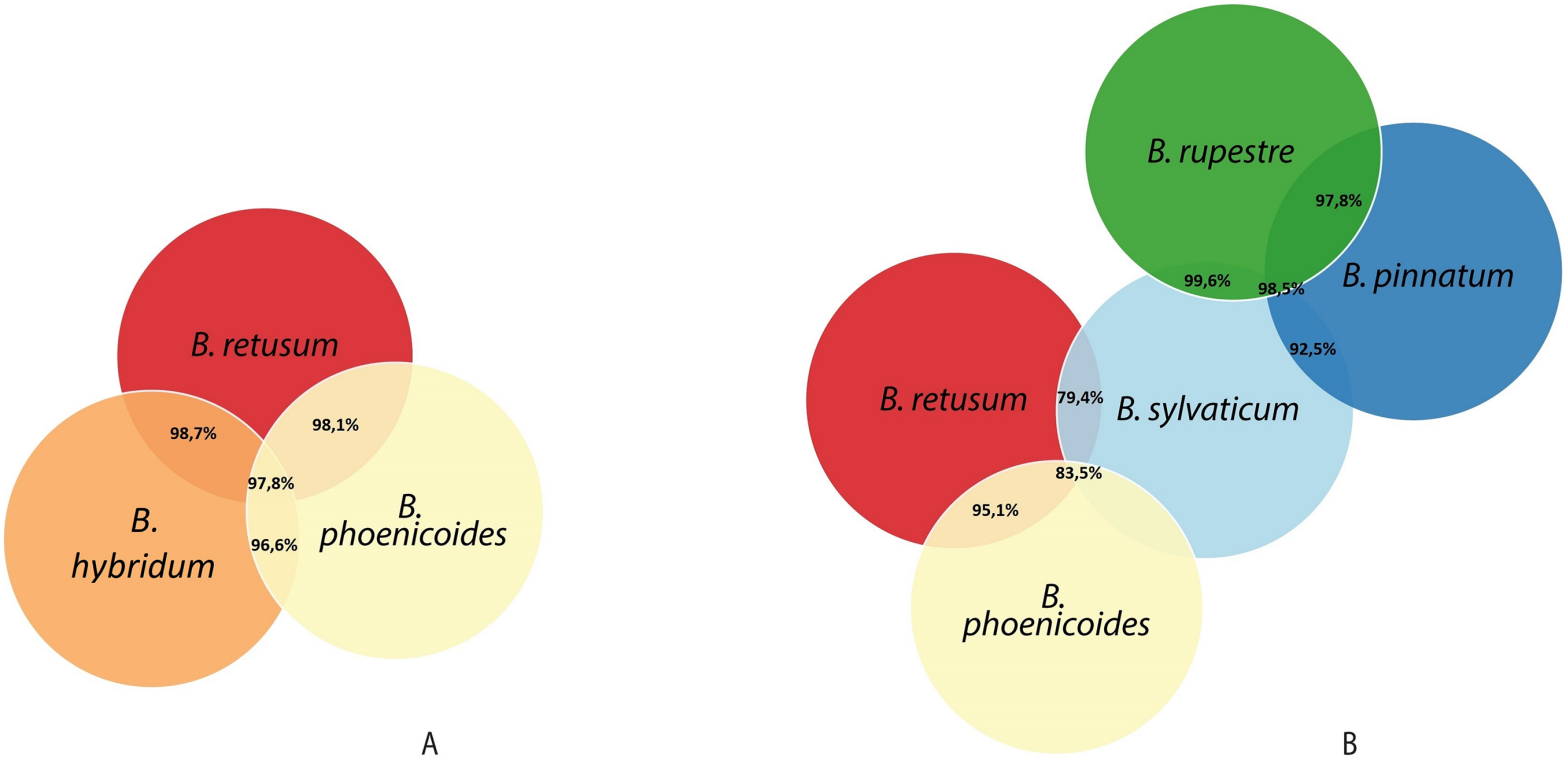
Co-occurrences found using a geographical distance of 20 km for the different species in (A) dataset 6 and (B) dataset 7.

Analyzing the correlation of all the species (dataset 7), only four of them presented positive co-occurrence relationships up to a distance of 10 km (*B. phoenicoides, B. retusum, B. pinnatum*, and *B. sylvaticum*), while *B. rupestre* appears only in 20 km, then it is not common to find *B. Rupestre* near other *Brachypodium* species in a distance lower than 10 km. Species with a large amount of records impacted the co-occurrence analysis, and only species that appeared in almost 15% of all transactions were considered with statistical significance to create positive rules by the algorithm. Figure 3 presents the case of *B. hybridum*, where in dataset 6 it appeared in 15.9% of the transactions, while in dataset 7 it was 3.9%; here, we must bear in mind that dataset 6 did not present the two species with the widest distribution, which can play an important role in the number of correlations that are established. Hence, *B. hybridum* lost statistical importance and this is the reason why it was not included in the positive rules created by dataset 7. We found an interesting behavior by *B. hybridum* as this species always appeared as antecedent in the generated rules (Fig. 2A), thus demonstrating its importance in creating co-occurrence relationships. After 20 km, complex rules were generated, which included more than two species in more than 50% of all rules, such as the case of dataset 7 (see Fig. 4). This is possible because the size of the study area increases and more species can be found within, thus creating more transactions. This was observed in the case of *B. sylvaticum* that has co-occurrence relations with many species such as *B. pinnatum*, *B. rupestre*, *B. retusum*, and *B. phoenicoides* (see Fig. 5), due to its broad distribution in Europe, Asia, and North of Africa. When *B. sylvaticum* and *B. pinnatum* were not included in the analysis, we found close relations between *B. hybridum*, *B. phoenicoides*, and *B. retusum* in a distance of 20 km, and almost 40% of those co-occurrences included three species, suggesting their importance (Fig. 5A).

Finally, we found that species with a restricted distribution (endemic) like *B. arbuscula* and *B. boissieri*, or particularly present in a continent or place as is the case of *B. flexum, B. bolusii* from African or *B. kawakami* from Taiwan, *B. madagascariense* from Madagascar, and *B. mexicanum* from American, they did not present correlations with other species, which was to be expected, helping us to corroborate that the statistics are correct.

This study can play an important role in the knowledge of the associations of the species, in a level of experimental ecology, improving our capacity to predict correlations in species from big spatial data. For example, Swenson & Jones (2017) clearly demonstrate that the bioinformatics and data mining techniques are vital to analyze large volumes of biological data that involves of plant ecology.

## Conclusions

Computer science and biology have merged to a relatively new discipline called bioinformatics, which can resolve biological problems using computational techniques (Arango-López et al., 2017). This interdisciplinary work is driven by the need to analyze and make sense of a large amount of data produced by biological systems and in this study proved to be useful for estimating co-occurrences using only georeferenced information, such as latitude and longitude. This study contributes to the understanding ecological establishment of different species and their association importance and will be helpful for future researches that require this information. We expect that the proposed data-mining method will be useful for when a priori knowledge is available and it aims to demonstrate the utility of public data (e.g., GBIF).

## Supplemental Information

10.7717/peerj.6193/supp-1 Supplemental Information 1 Generated rules.

10.7717/peerj.6193/supp-2 Supplemental Information 2 Positive-generated rules.

## Additional Information and Declarations

### Data Availability

The following information was supplied regarding data availability: Code is available in our GitHub repository here: https://github.com/simonorozcoarias/co-ocurrence_analysis. Raw data is available in GitHub: https://github.com/simonorozcoarias/co-ocurrence_analysis/tree/master/raw_data.
